# Histone Deacetylase HDAC4 Promotes Gastric Cancer SGC-7901 Cells Progression via p21 Repression

**DOI:** 10.1371/journal.pone.0098894

**Published:** 2014-06-04

**Authors:** Zhen-Hua Kang, Chun-Yan Wang, Wen-Liang Zhang, Jian-Tao Zhang, Chun-Hua Yuan, Ping-Wei Zhao, Yu-Yang Lin, Sen Hong, Chen-Yao Li, Lei Wang

**Affiliations:** 1 Department of Colorectal and Anus Surgery, First Hospital, Jilin University, Changchun, P. R. China; 2 The Tumor Research Institute of JiLin Province, Changchun, P. R. China; Peking University Health Science Center, China

## Abstract

Gastric cancer (GC) is one of the leading causes of cancer death in the world. The role of histone deacetylase 4 (HDAC4) in specific cell and tissue types has been identified. However, its biological roles in the development of gastric cancer remain largely unexplored. Quantitative real time PCR (qRT-PCR) and western blot were used to analyze the expression of HDAC4 in the clinical samples. siRNA and overexpression of HDAC4 and siRNA p21 were used to study functional effects in a proliferation, a colony formation, a adenosine 5′-triphosphate (ATP) assay and reactive oxygen species(ROS) generation, cell cycle, cell apoptosis rates, and autophagy assays. HDAC4 was up-regulated in gastric cancer tissues and several gastric cancer cell lines. The proliferation, colony formation ability and ATP level were enhanced in HDAC4 overexpression SGC-7901 cells, but inhibited in HDAC4 knockdown SGC-7901 cells. HDAC4 knockdown led to G0/G1 phase cell arrest and caused apoptosis and ROS increase. Moreover, HDAC4 was found to inhibit p21 expression in gastric cancer SGC-7901 cells. p21 knockdown dramatically attenuated cell proliferation inhibition, cell cycle arrest, cell apoptosis promotion and autophagy up-regulation in HDAC4-siRNA SGC-7901 cells. We demonstrated that HDAC4 promotes gastric cancer cell progression mediated through the repression of p21. Our results provide an experimental basis for understanding the pro-tumor mechanism of HDAC4 as treatment for gastric cancer.

## Introduction

Gastric cancer (GC) is the fourth most common malignancy and the second leading cause of cancer death worldwide [1]. Asian and South American countries have a higher incidence rate of GC than the United States and Western Europe [2]. Most targeted therapies focus on vascular endothelial growth factor (VEGF) and epidermal growth factor receptor (EGFR) related indications in advanced GC. Compounds against novel targets, such as mTOR [3], c-Met (hepatocyte growth factor receptor) [4], and HDACs, are also under investigation.

Histone deacetylase 4 (HDAC4), which is a class IIa HDAC, is known to exist in distinct transcriptional corepressor complexes. HDAC4 is a critical component of the DNA damage response pathway that acts through 53BP1 [5]. HDAC4 represses the expression of the cyclin-dependent kinase inhibitor p21 (also known as p21WAF1/Cip1) in human cancer cells through an Sp1-dependent, p53-independent mechanism [6]. HDAC4 promotes the growth of colon cancer cells via repression of p21 [7]. Inactivation of HDAC4 by small interfering RNA or HDAC inhibitors suppresses neuronal cell death [8].

The role of HDAC4 in specific cells (HeLa (cervical cancer), U373 (glioma), OVCAR (ovarian), T98G (glioma), HCT116 (colorectal), NHA (astrocytic) and SKBR3 (breast)) and tissues (cerebral cortex, testis, prostate, and the epidermal layer of the skin) is becoming more clear [9]. However, the exact mechanism of HDAC4 in gastric cancer has not been determined. Therefore, the purpose of this study was first to evaluate the expression levels of HDAC4 in human gastric cancer tissues and cell lines. Second, the effect of HDAC4 on gastric cancer cell lines was examined using overexpression and knockdown. Finally, we investigated whether HDAC4 had a relationship with p21 in gastric cancer cells. Together, our aim was to find whether HDAC4 has a biological role in GC development and to elucidate the underlying mechanism.

## Materials and Methods

### Tissue samples

Twenty-nine paired primary gastric carcinoma tissues and distant normal gastric tissues were collected from patients (age: 58.46±9.17 years) during routine therapeutic surgery in the Department of Gastrointestinal Surgery, the First Hospital of Jilin University. All samples were obtained with informed consent according to the Declaration of Helsinki and approved by the Human Ethics Committee of the First Hospital of Jilin University and Jilin University, PR China. Written informed consent was obtained from all participants.

### Cell lines and culture

The human gastric cancer cell lines SGC-7901, AGS, and BGC-823 and the normal gastric epithelium cell line GES were obtained from American Type Culture Collection (Manassas, VA) and cultured in RPMI 1640 medium with 10% fetal bovine serum in a humidified atmosphere of 5% CO_2_ at 37°C.

### Stable transfected cell line and transient transfection

The full-length HDAC4 fragment was amplified by reverse transcription PCR from GES cells using the specific primers as previously described [10] and inserted into the BamH I and Hind III sites of pcDNA3.1(+) (Clontech, Hampshire, UK). The pcDNA3.1 (+)-HDAC4 plasmid was verified by sequence analysis to confirm the absence of mutations. SGC-7901 cells were seeded in 6-well plates and transfected with the pcDNA3.1 (+)-HDAC4 and pcDNA3.1 (+)-vectors, respectively, using Lipofectamine 2000 (Invitrogen, Carlsbad, CA) according to the instructions provided by the manufacturer for 24 h without antibiotic selection. Then, the cells were cultured in medium containing 400 µg/ml G418 (Sigma, St. Louis, MO) until all of the cells in the non-transfected control culture were killed.

SGC-7901 cells were seeded on 6-well plates and then transfected with non-targeting siRNA or siRNA directed against human HDAC4 or p21 (Santa Cruz) using Lipofectamine 2000 according to the instructions provided by the manufacturer. Stretch experiments were performed on cells 48 or 72 h post-transfection.

### RNA isolation and quantitative real time PCR (qRT-PCR)

Total RNA was isolated from tissues or cells by TRIzol reagent (Invitrogen), and reverse transcriptions were performed using the Takara RNA PCR kit (Takara, Dalian, China) following the manufacturer's instructions. Quantitative PCR was performed using a SYBR Green Premix Ex Taq (Takara, Japan) in a real-time PCR system (Eppendorf, Germany). The relative expression ratio in each paired tumor and non-tumor tissue was calculated by the 2^−ΔΔCT^ method.

### Cell counting kit-8 (CCK-8) assay

The proliferations of the SGC-7901 cells were assessed using a CCK-8 assay (Beyotime, Jiangsu, China) according to the protocol recommended by the manufacturer. Briefly, the cells were cultured in a 96-well plate at a concentration of 2000 cells per well. At 0, 1, 2 and 3 days after transfection, the cell proliferation assay was performed by the addition of 10 µl of CCK8 solution to each well, followed by incubation at 37°C for 2 h. Absorbance was measured by an ELISA reader at a wavelength of 450 nm.

### Colony formation assay

Briefly, the HDAC4-overexpressing and -knockdown SGC-7901 cells were seeded in triplicate (1,000 cells per 60 mm culture dish) and incubated at 37°C for two weeks to form clones. The cells were washed with PBS, fixed with 4% paraformaldehyde for 15 min, and stained with crystal violet (0.5% crystal violet, 1% paraformaldehyde and 20% methanol in PBS) for 30 min. The colonies on each plate were counted, and cell survival was expressed as a percentage of the number of surviving colonies on the control plates.

### Cell cycle analysis

The cells were harvested and gently resuspended into single cell suspensions in Fluorescence Activated Cell Sorting (FACS) buffer (PBS containing 2% FBS). The cells were washed with PBS twice and fixed in cold 70% ethanol overnight. Cells were then washed twice with cold PBS, resuspended in RNase A solution and incubated for 30 min at 4°C. The suspension was added to 0.05 mg/ml propidium iodide (Beyotime) followed by incubation at 4°C for 30 min and analysis using FACSCalibur (BD Biosciences, San Jose, CA).

### Cell apoptosis assay

Propidiumiodide (PI, 1 µg/ml) and Annexin V-FITC (1 µg/ml) were used for the determination of cell apoptosis. Briefly, the cells were trypsinized and incubated with PI and Annexin V-FITC for 15 min at 37°C. The samples were detected using a FACS Calibur flow cytometer. All of the experiments were performed in triplicate.

### Intracellular adenosine 5′-triphosphate (ATP) levels assay

Intracellular ATP levels were assayed using bioluminescence [11]. Briefly, cells were lysed and centrifuged at approximately 15,000 g for 10 min at 4°C. The supernatants were then mixed with an ATP assay mix working solution (Sigma), and the amount of light emitted was immediately measured with a luminometer (Thermo Scientific Luminoskan Ascent, Walthan, MA). The luminescence data were normalized by the sample protein amounts (measured using a BCA assay).

### Reactive oxygen species (ROS) measurement

Generation of intracellular ROS was examined using 6-carboxy-2′,7′-dichlorofluorescein diacetate (H_2_DCFDA). Briefly, 5×10^4^ cells were plated in 60-mm dishes and allowed to attach overnight. The cells were stained with 5 µM H_2_DCFDA for 30 min at 37°C and then collected, and the fluorescence was analyzed with a fluorescence spectrometer (Flex StationII 384, Molecular Devices) at 485 nm (excitation) and 538 nm (emission). Cellular oxidant levels were expressed as relative DCF fluorescence per sample protein amounts (BCA method). In some experiments, cells were pretreated with 10 mM *N*-acetylcysteine (NAC) prior to the analysis of ROS generation.

### Western blot analysis

Cells were lysed in IP lysis buffer (Beyotime), denatured for 10 min at 95°C, sheared with an insulin syringe, and resolved on SDS/PAGE gels. After immunoblotting, the membranes were blocked and incubated with primary antibodies in PBS/0.1% Tween-20 with 5% nonfat dry milk. Antibodies directed against HDAC4, p21, LC3, Beclin 1, Atg 7, Caspase 3, 9, Bcl-2, Bax and anti-β-Actin were all purchased from Santa Cruz Biotechnology (Santa Cruz). Chemiluminescent detection was assayed using an ECL detection kit (Pierce, Rockford, IL, USA). The results were analyzed using Quantity One software to obtain the optical density ratio of target protein to β-Actin.

### Immunofluorescence

The cells were plated on coverslips, fixed with 4% paraformaldehyde (Sigma-Aldrich) for 10 min and permeabilized with 0.1% Triton X-100/PBS. The cells were blocked with 1% BSA for 1 h, followed by incubation for overnight at 4°C with the anti-LC3 antibody, and then cells were incubated with FITC-conjugated secondary antibody (Beyotime) for 1 h. The nuclei were stained with 4,6-diamidino-2-phenylindole (DAPI; Sigma). The fluorescence imaging was visualized using a confocal laser scanning microscope (Carl Zeiss, Oberkochen, Germany).

### Statistical analysis

All data are representative of at least three independent experiments. The data are presented as the means ± S.E.M. Statistical significance was calculated by one-way analysis of variance (ANOVA) or by Student's t-test between the two groups using GraphPad Prism 5 statistical software. *P* values <0.05 were considered significant.

## Results

### HDAC4 expression were up-regulated in gastric cancer tissues and cell lines

**Figure 1:**
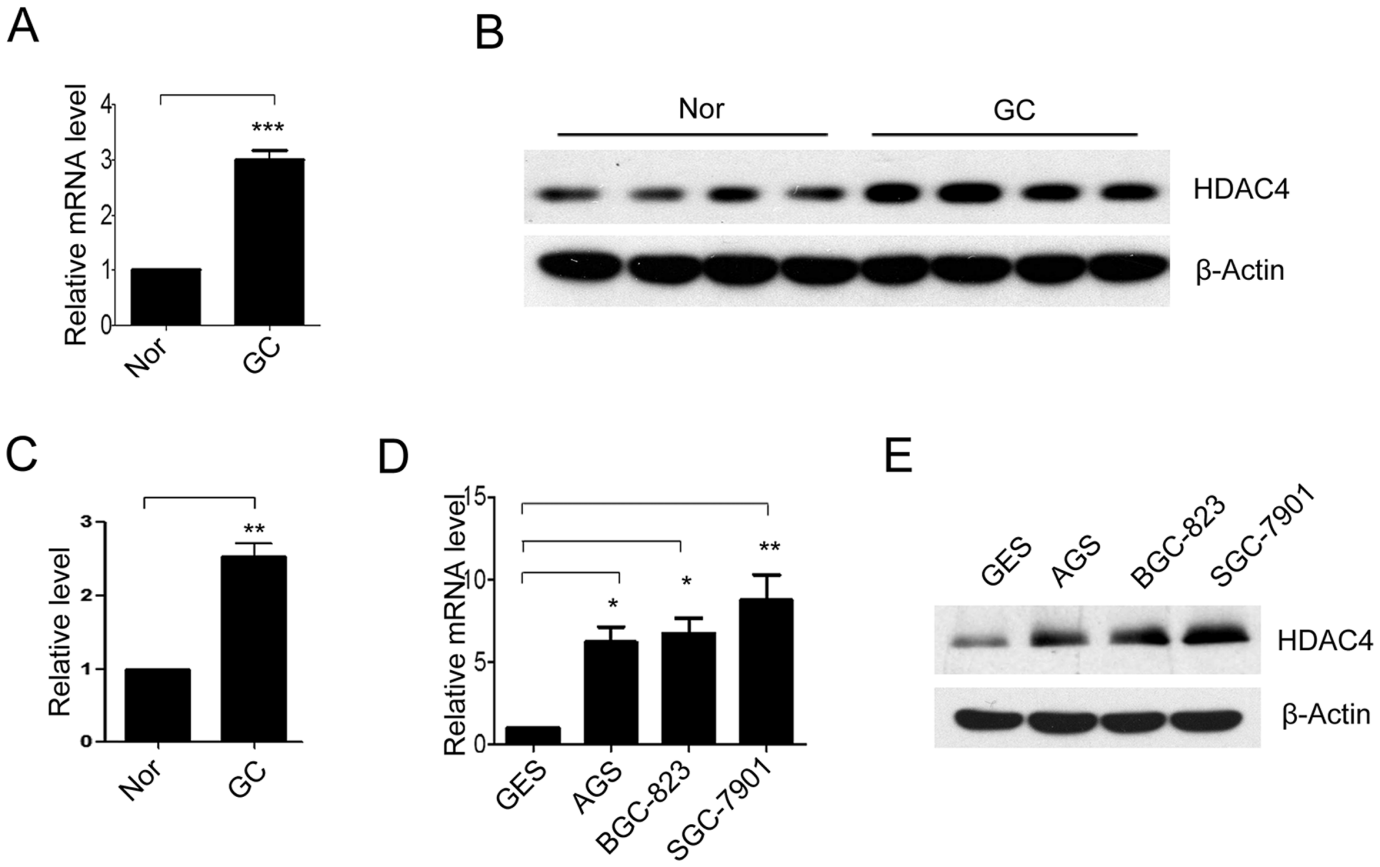
The expression of histone deacetylase 4 (HDAC4) in gastric cancer tissues and cell lines. The mRNA and protein levels of HDAC4 were analyzed by quantitative real-time PCR (qRT-PCR) and western blot in normal tissues (Nor) or gastric cancer tissues (GC) (**A**) and (**B**) (n = 29). Densitometric analysis of HDAC4 in Fig. 1B (**C**). The mRNA and protein levels of HDAC4 were analyzed in normal gastric epithelium cells (GES cells) and several gastric cancer cell lines, including AGS, BGC-823 and SGC-7901 cells (**D**) and (**E**) (n = 4). Data were expressed as mean ± S.E.M. *P<0.05, **P<0.01, ***P<0.001.

First, we analyzed HDAC4 expression in twenty-nine paired gastric cancer and adjacent non-tumor tissues by qRT-PCR analysis and western blot. We found that HDAC4 was significantly up-regulated in gastric carcinoma tissue (Figures 1A, B and C, **P<0.01, ***P<0.001). Additionally, several gastric cancer cell lines were also analyzed. We observed higher expression of HDAC4 in three gastric cancer cell lines (AGS, BGC-823 and SGC-7901), compared with a normal gastric epithelium cell line (GES) (Figures 1D and E, *P<0.05, **P<0.01). Therefore, our results demonstrated that HDAC4 expression were up-regulated in both gastric cancer tissues and cell lines.

### The HDAC4 expression was successfully down- or up-regulated in SGC-7901 cells

**Figure 2:**
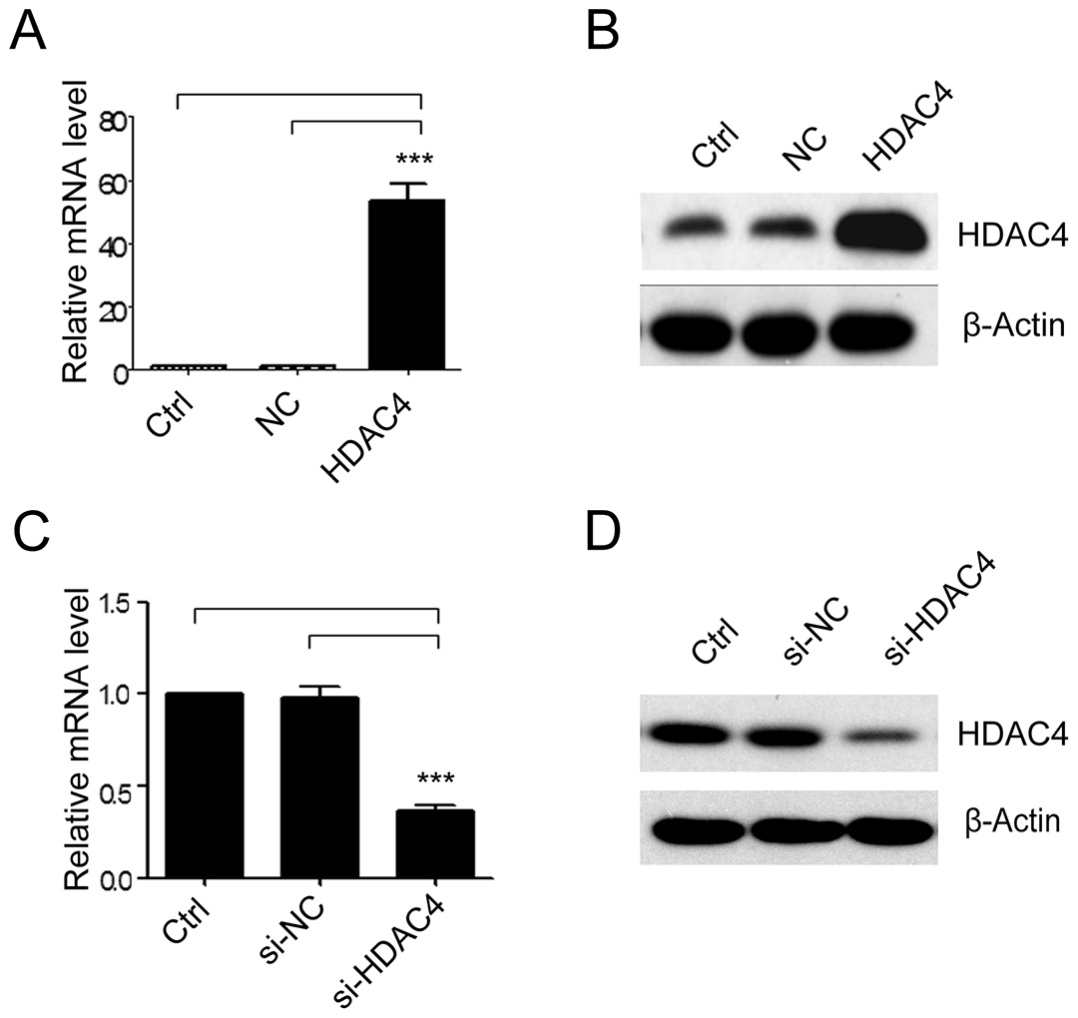
The expression of HDAC4 in transfected SGC-7901 cells. HDAC4 expression was determined in SGC-7901 cells transfected with empty vector (NC) or HDAC4 (**A**) and (**B**). HDAC4 expression was determined in SGC-7901 cells transfected with siRNA oligos targeting HDAC4 (si-HDAC4) or scrambled siRNA (si-NC) by qRT-PCR and western blot (**C**) and (**D**) (n = 4). Data were expressed as mean ± S.E.M. ***P<0.001.

A pcDNA3.1(+) expression vector was used to overexpress HDAC4 in SGC-7901 cells in order to examine its effectiveness as a growth regulator. After stable transfection, the expression of HDAC4 mRNA levels was more abundant in the pcDNA3.1(+)-HDAC4 cells than in the non-transfected cells or negative control (NC) SGC-7901 cells (Figure 2A, ***P<0.001), which was consistent with result of western blot analysis (Figure 2B).

To evaluate the gene-silencing efficacy of human HDAC4-siRNA, the extent of HDAC4 protein expression was measured after HDAC4-siRNA transfection and a 48-h incubation period in human gastric cancer cell line SGC-7901. Real-time PCR analysis showed that HDAC4 siRNA infection resulted in 36.3% knockdown of HDAC4 mRNA levels (Figure 2C, ***P<0.001). Western blot analysis showed that the HDAC4 protein was significantly reduced (Figure 2D), consistent with its mRNA reduction. Taken together, these data suggested that HDAC4 siRNA could significantly suppress the endogenous HDAC4 expression.

### HDAC4 promoted cell proliferation and colony formation

**Figure 3:**
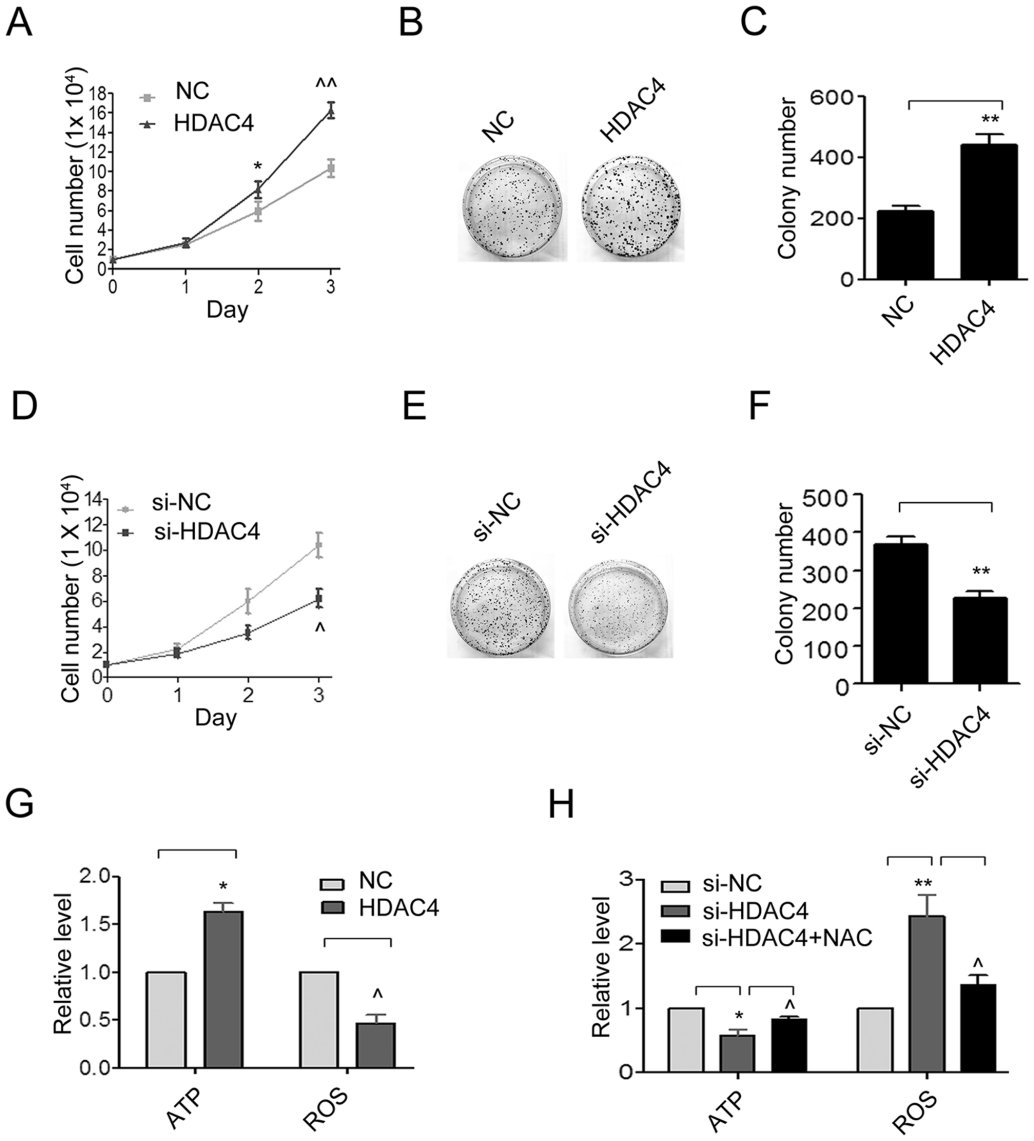
Roles of HDAC4 on SGC-7901 cell proliferation and colony formation. The growth curve (*P<0.05 and *∧∧P<*0.01 compared with NC group) (**A**), clone formation (**B**) and (**C**) and the relative ATP level and ROS generation (**G**) in SGC-7901 cells transfected with empty pcDNA3.1(+)-vector (NC) or HDAC4. The growth curve (*∧P<*0.05 compared with si-NC group) (**D**), clone formation (**E**) and (**F**) in SGC-7901 cells transfected with siRNA oligos targeting HDAC4 (si-HDAC4) or with scrambled siRNA oligo (si-NC). The effect of the antioxidant *N*-acetylcysteine (NAC) on relative ATP level and ROS generation in SGC-7901 cells transfected with si-NC or si-HDAC4 (**H**). Data were expressed as mean ± S.E.M. *P<0.05, **P<0.01, *∧P<*0.05, *∧∧P<*0.01.

Next, we examined the effect of HDAC4 on cell growth. Cell proliferation was examined using Cell Counting kit-8 (CCK-8) at the time points of 24, 48 and 72 h. The growth curves showed that when HDAC4 was overexpression in SGC-7901 cells, the cell growth was increased by 1.5 fold in comparison with control group on day 3 (Figure 3A, *P<0.05, *∧∧P*<0.01). In addition, the colony formation assay displayed a dramatic increase of 2 fold in colony number when SGC-7901 cells were transfected with the pcDNA3.1(+)-HDAC4 relative to the NC group (Figures 3B and C, **P<0.01).

We also examined the effects of HDAC4 down-regulation in SGC-7901 cells. The CCK-8 assay and colony formation assay showed that the HDAC4 down-regulation suppressed the proliferation ability (Figure 3D, *∧P*<0.05) and the colony formation numbers of SGC-7901 cells (Figures 3E and F, **P<0.01). In summary, these data suggested that HDAC4 might be a growth promoter in SGC-7901 cells.

### HDAC4 increased ATP levels and decreased ROS generation

An increase in the ATP levels in HDAC4 overexpressing cells was observed compared to the NC SGC-7901 cells (Figure 3G, *P<0.05). Moreover, the ATP level was decreased in HDAC4 knockdown cells (Figure 3H, *P<0.05).

Because intracellular ROS generation may be related to mitochondrial dysfunction, we further examined whether HDAC4 could stimulate ROS generation in SGC-7901 cells. The results demonstrate that a significant reduction of ROS generation was observed in pcDNA3.1(+)-HDAC4 SGC-7901 cells compared to NC SGC-7901 cells (Figure 3G, *∧P*<0.05). Meanwhile, silencing of HDAC4 robustly activated ROS generation in SGC-7901 cells (Figure 3H, **P<0.01). Blocking ROS production using the antioxidant NAC significantly inhibited ROS generation (Figure 3H, *∧P*<0.05). This blocking of ROS generation by pretreatment of the cells with NAC also markedly prevented ATP loss in HDAC4-siRNA SGC-7901 cells (Figure 3H, *∧P*<0.05).

### The down-regulated HDAC4 expression arrested cells in G0/G1 phase

**Figure 4:**
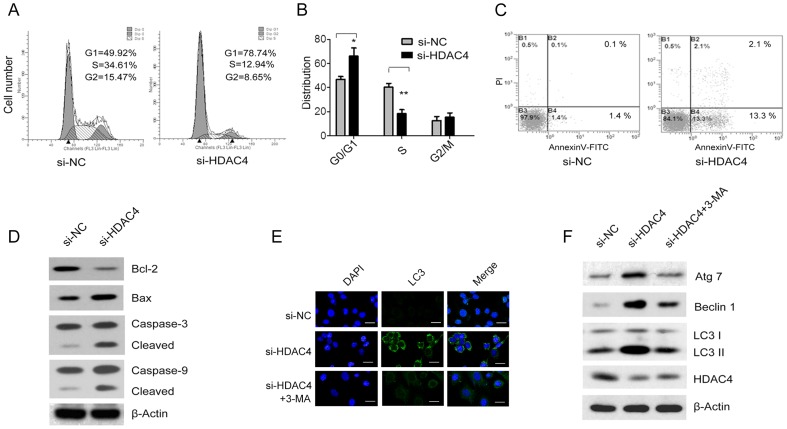
Roles of HDAC4 knockdown on SGC-7901 cell cycle, apoptosis and autophagy. Flow cytometry analysis depicted cell cycle progression of SGC-7901 cells after knockdown of HDAC4 (**A**) and the cell cycle profiles were analyzed to quantitate cell cycle distribution (**B**). The SGC-7901 cells transfected with scrambled control (si-NC) or HDAC4 siRNA oligos (si-HDAC4) apoptosis was evaluated by flow cytometry using Annexin V-FITC/PI (**C**). Expression of pro- and anti-apoptotic proteins and caspases 3 and 9 were assayed by western blot (**D**). The cells were immunostained with anti-LC3 antibodies (FITC, green) and nuclei were stained with DAPI (blue) and analyzed by confocal microscopy (**E**). Western blot analysis of Atg7, Beclin 1 and LC3 protein expression levels in SGC-7901 cells transfected with scrambled control (si-NC) or HDAC4 siRNA oligos (si-HDAC4) treated with or not with 3-MA (**F**). Data were expressed as mean ± S.E.M. *P<0.05, **P<0.01.

The down-regulation of HDAC4 exhibited a clear increase in the proportion of cells in the G0/G1 phase (78.74% compared with 49.92% in the NC-siRNA group). There was also a corresponding decrease in the number of cells in the S phase (12.94% compared with 34.61% in the NC-siRNA group) (Figure 4A). The quantitate cell cycle distribution results were shown that HDAC4 knockdown significantly induced SGC-7901 cells G0/G1 arrest and S phage inhibition (Figure 4B, *P<0.05, **P<0.01). Hence, these findings suggest that the HDAC4 level could regulate cell cycle progression.

### The down-regulated HDAC4 expression induced apoptosis and autophagy

**Figure 5:**
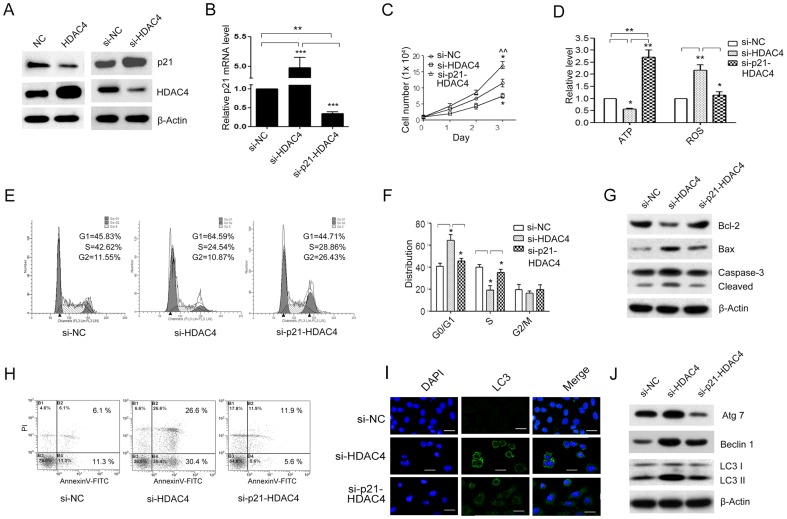
p21 knockdown reversed the effect of down-regulated HDAC4 on the inhibition of SGC-7901 cell growth. Expression of p21 was analyzed by western blot in SGC-7901 cells transfected with empty pcDNA3.1(+)-vector (NC) or HDAC4 and scrambled siRNA control (si-NC) or HDAC4 siRNA oligos (si-HDAC4) respectively (**A**). The p21 mRNA level was analyzed by qRT-PCR in SGC-7901 cells transfected with si-NC, si-HDAC4 alone or combination with siRNA p21 (si-p21) (**B**). The cell growth curve was measured by CCK-8 assay (*P<0.05 compared with si-NC group, *∧∧P<*0.01 compared with si-HDAC4 group) (**C**). The relative ATP levels and ROS generation (**D**). The cell cycle analysis (**E**, **F**). Apoptosis was assayed by western blot (**G**) and flow cytometry using Annexin V-FITC/PI (**H**) respectively. Autophagy was assessed by immunofluorescence (**I**) and western blot (**J**) respectively in SGC-7901 cells transfected with si-NC, si-HDAC4 alone or combination with si-p21. Data were expressed as mean ± S.E.M. *P<0.05. **P<0.01, ***P<0.001.

To study whether the down-regulated HDAC4-induced cell growth inhibition was related to cell apoptosis, the effect of down-regulated HDAC4 on cell apoptosis was evaluated by flow cytometry using Annexin V-FITC/PI double staining. It was observed that apoptosis increased markedly in HDAC4-siRNA SGC-7901 cells compared with the NC-siRNA group (Figure 4C). We further confirmed the induction of apoptosis through the activation of caspase-3 and 9 by western blot. The analysis revealed that down-regulated HDAC4 increased cleavage of caspases-3 and 9 compared with NC-siRNA group. The expression of the anti-apoptotic protein Bcl-2 and the pro-apoptotic protein Bax were also quantified. The Bax/Bcl-2 ratio was significantly increased in HDAC4-siRNA SGC-7901 cells when compared to the NC-siRNA group (Figure 5D).

To investigate whether down-regulated HDAC4 induced autophagy in SGC-7901 cells, we first examined the intracellular localization of LC3 in HDAC4-siRNA SGC-7901 cells by immunofluorescence analysis using fluorescent antibodies to LC3. The specific punctuate distribution of endogenous LC3 were observed as punctate dots of green fluorescence in HDAC4-siRNA SGC-7901 cells compared to that of NC-siRNA group (Figure 4E), indicating that autophagy was induced as a means of survival. The subcellular distribution of LC3 were significantly inhibited by the autophagy-specific inhibitor 3-MA in HDAC4-siRNA SGC-7901 cells (Figure 4E). Then, the autophagy related proteins Atg7, Beclin 1 and the ratio of LC3-II to LC3-I were analyzed by western blot. We observed that the expression levels of Atg7, Beclin 1 and LC3-II were all significantly increased in HDAC4-siRNA SGC-7901 cells compared with the NC-siRNA group (Figure 4F). The Atg7, Beclin 1 and the ratio of LC3-II to LC3-I decreased markedly in HDAC4-siRNA SGC-7901 cells that were treated with 3-MA compared with the NC-siRNA group (Figure 4F).

### The down-regulated HDAC4 expression inhibited cell growth through p21 up-regulation

Because the treatment of human cancer cells with HDAC inhibitors consistently leads to up-regulation of p21 expression, a cyclin-dependent kinase inhibitor that is a well-established target of HDAC inhibitors [6], [7], [12], we sought to determine whether overexpression or knockdown of HDAC4 could affect p21 regulation and to speculate the mechanism of HDAC4-mediated growth promotion in gastric cancer cells.

As shown in Figure 5A, the up-regulation of HDAC4 dramatically led to the decreased p21 protein expression. In contrast, HDAC4 down-regulation led to the increased p21 expression (Figure 5A). We then examined whether p21 knockdown could affect cell growth and apoptosis in SGC-7901 cells in which HDAC4 was deleted. The SGC-7901 cells were co-transfected with siRNA HDAC4 and siRNA p21. The p21 mRNA level was significantly decreased in HDAC4-siRNA SGC-7901 cells after p21 knockdown (Figure 5B, **P<0.01, ***P<0.001). p21 knockdown dramatically attenuated cell proliferation inhibition in HDAC4-siRNA SGC-7901 cells (Figure 5C, *P<0.05, ∧∧*P*<0.01). The ATP level was increased, but intracellular ROS generation decreased in siRNA-p21-HDAC4 group compared with the siRNA HDAC4 group (Figure 5D, *P<0.05, **P<0.01). p21 down-regulation significantly attenuated G0/G1 arrest and S phage inhibition in HDAC4-siRNA SGC-7901 cells (Figures 5E and F, *P<0.05). Immunoblotting showed that the amount of Bcl-2 was significantly higher, but Bax was lower in siRNA-p21-HDAC4 cells (Figure 5G). The cell apoptosis decreased markedly in siRNA-p21-HDAC4 SGC-7901 cells compared with the siRNA HDAC4 group (Figure 5H). It was also observed that p21 knockdown could rescue the increased autophagy punctuated fluorescent spots (Figure 5I) and Atg 7, Beclin 1 and LC3II proteins expression in SGC-7901 cells induced by siRNA HDAC4 (Figure 5J). Collectively, these data indicated that down-regulation of p21 could mimic the effect of HDAC4 overexpression and might be an important mediator in HDAC4-mediated SGC-7901 cell growth promotion.

## Discussion

Numerous HDAC inhibitors, which have been shown to inhibit proliferation and induce differentiation or apoptosis in tumor cells, are being investigated in clinical trials either as anti-cancer agents or in conjunction with other treatment [13]. High expression of HDAC1/2 was found in gastric carcinoma tissues [14]. In our study, we showed that HDAC4 expression were up-regulated in gastric cancer tissues and cell lines *in vitro*. HDAC4 down-regulation in SGC-7901 cells suppressed the cell proliferation and the colony formation numbers, arrested cell cycle and induced cell apoptosis dependent on the activation of caspases 3 and 9, which is consistent with the anti-proliferative and proapoptotic effects of HDAC inhibitors in human cancer cell lines [15] and with the previously reported observation that HDAC4 down-regulation reduces clonogenic survival and induces apoptosis in HeLa cells [5]. The proproliferative effect of HDAC4 in gastric cancer cells is consistent with that reported previously for the class I HDACs, HDAC1, -2, and -3 [16]. Thus, targeting the inhibition of HDAC4 activity may be a potential therapeutic approach to treat gastric cancer.

Autophagy is essentially a protein degradation system of the cell's own lysosomes. A variety of stress signals, such as nutrient starvation or treatment with different anticancer agents, stimulate the autophagy process [17]. Autophagy is generally characterized by the presence of autophagosomes and an increase in cleavage of LC3 [18]. The role of autophagy in the process of cell death is controversial, but it has been confirmed that crosstalk between apoptosis and autophagy is essential in programmed cell death [19]. In our study, the induction of autophagy in HDAC4-siRNA SGC-7901 cells was evidenced by the punctuate pattern of LC3 immunostaining and the accumulation of biochemical hallmark proteins of autophagy (Atg7, Beclin 1 and LC3) by western blot analysis. Autophagy inhibition by the inhibitor 3-MA attenuated the autophagy induction in HDAC4-siRNA SGC-7901 cells. Thus, we speculated autophagy occurred may protect SGC-7901 cancer cells apoptosis induced by HDAC4 knockdown.

p21 protein, also known as cyclin-dependent kinase (CKD) inhibitor 1, regulates the G1 phase progression of the cell cycle. p21 is often mis-regulated in human cancers, and it can act as a tumor suppressor or as an oncogene [20]. Those with p21-positive and p53-negative cancers have significantly higher survival curves in gastric carcinoma [21], whereas the loss of p21 expression along with increased p53 detection is associated with poor prognosis and decreased overall survival in gastric cancer [22]. Consistent with the result that p21 protein was down-regulated in the gastric cancer tissues [23], we observed that HDAC4 promotes gastric cancer cell proliferation and growth, mediated by the repression of p21 in gastric cancer cells, and is accompanied by an increase in ATP levels and repression of ROS generation. Therefore, we explored whether p21 was an important mediator for HDAC4-mediated SGC-7901 cell growth promotion. In this study, HDAC4 knockdown significantly induced the increased expression of p21 protein, while HDAC4 overexpression significantly decreased the expression of p21 protein in SGC-7901 cells. These data implied that p21 might be a downstream target of HDAC4. Functional analyses showed down-regulation of p21 could mimic the effect of HDAC4 overexpression on SGC-7901 cell growth promotion. Taken together, these results suggested that HDAC4 down-regulation could promote SGC-7901 cell apoptosis by up-regulating p21 expression. p21 might be a tumor suppressor in the development and progression of gastric cancer, the expression of which may aid in controlling a variety of malignant behaviors of gastric cancer. However, the potential relevance of HDAC4 regulation of p21 expression also needs to be viewed in the context that there are multiple key factors and pathways that potentially modulate the expression of p21 in gastric cancer.

Taken together, our findings have revealed an important role for HDAC4 in controlling human gastric cancer cell line SGC-7901 development via regulation of p21, suggesting that alteration of HDAC4 expression and/or activity may be an important event during gastric cancer. In conclusion, these findings identify HDAC4 as an important regulator of proliferation of gastric cancer through repression of p21 *in vitro*.
